# Treatment With a Soluble Bone Morphogenetic Protein Type 1A Receptor (BMPR1A) Fusion Protein Increases Bone Mass and Bone Formation in Mice Subjected to Hindlimb Unloading

**DOI:** 10.1002/jbm4.10012

**Published:** 2017-10-09

**Authors:** Frank C. Ko, Miranda Van Vliet, Rachel Ellman, Daniel Grasso, Daniel J Brooks, Jordan M Spatz, Chrissy Conlon, J Ignacio Aguirre, Thomas J Wronski, Mary L Bouxsein

**Affiliations:** ^1^ Center for Advanced Orthopedic Studies Beth Israel Deaconess Medical Center Boston MA USA; ^2^ Endocrine Unit Massachusetts General Hospital Boston MA USA; ^3^ Massachusetts Institute of Technology (MIT) Health Sciences and Technology Program Cambridge MA USA; ^4^ Department of Physiological Sciences University of Florida Gainesville FL USA; ^5^ Department of Orthopedic Surgery Harvard Medical School Boston MA USA

**Keywords:** BMPS/TGF‐βS, BONE HISTOMORPHOMETRY, BONE QCT/μCT, BIOMECHANICS, PRECLINICAL STUDIES

## Abstract

Previous work has shown that the soluble murine BMPR1A–fusion protein (mBMPR1A‐mFc) binds to BMP2 and BMP4 with high affinity, preventing downstream signaling. Further, treatment of intact and ovariectomized mice with mBMPR1A‐mFc leads to increased bone mass, and improved bone microarchitecture and strength, via increased bone formation and reduced resorption. In this study, we tested the effects of mBMPR1A‐mFc on disuse‐induced bone loss caused by 21 days of hindlimb unloading (HLU) via tail suspension versus cage controls (CONs). Adult female C57BL/6J mice (12 weeks old) were assigned to one of four groups (*n* = 10 each): CON‐VEH; CON‐mBMPR1A‐mFc; HLU‐VEH; and HLU‐mBMPR1A‐mFc. Mice were injected subcutaneously with VEH or mBMPR1A‐mFc (4.5 mg/kg, 2×/week). Leg BMD declined in the HLU‐VEH group (–5.3% ± 1.3%), whereas it was unchanged in HLU‐mBMPR1A‐mFc (–0.3% ± 0.9%, *p* < 0.05 versus HLU‐VEH). Leg BMD increased significantly more in CON‐mBMPR1A‐mFc than CON‐VEH (10.2% ± 0.6% versus 4.4% ± 0.8%). In the femur, trabecular, and cortical bone microarchitecture was worse in the HLU‐VEH compared to CON‐VEH mice, whereas mBMPR1A‐mFc treatment for 3 weeks led to greater Tb.BV/TV, Tb.Th, and midshaft Ct.Th in both the HLU and CON groups compared to comparable VEH‐treated counterparts (*p* < 0.05). HLU‐mBMPR1A‐mFc mice also had 21% greater failure load (*p* < 0.05) compared to their VEH‐treated counterparts. Dynamic histomorphometry indicated that treatment with mBMPR1A‐mFc led to significantly greater mineralizing surface and mineral apposition rate, resulting in a 3.5‐fold and fivefold higher bone formation rate in the mBMPR1A‐mFc‐treated CON and HLU animals versus VEH groups, respectively. mBMPR1A‐mFc‐treated mice had a similar osteoblast surface but significantly lower osteoclast surface than VEH‐treated animals in both the CON and HLU groups. Altogether, these findings suggest that treatment with the soluble BMPR1A fusion protein may be useful for maintenance of skeletal integrity in the setting of disuse‐induced bone loss. © 2017 The Authors *JBMR Plus* published by Wiley Periodicals, Inc. on behalf of American Society for Bone and Mineral Research.

## Introduction

Mechanical loading is essential to skeletal health, because muscle and bone atrophy occur in the setting of disuse. This disuse osteoporosis results in bone loss rates of 1% to 2% per month at weight bearing sites in those exposed to prolonged periods of reduced mechanical loading, such as occurs with paralysis, bed rest during recovery from injury or illness, or spaceflight.^1^ Current treatments for prevention of disuse osteoporosis rely on anti‐resorptive agents that lower the rate of bone turnover, decreasing both resorption and formation over time. Further, although bisphosphonate therapy inhibits bone loss in astronauts^2^ and in patients with acute and chronic spinal cord injuries,^3^, ^4^, ^5^ there is limited evidence of increased BMD, as is normally seen in postmenopausal women treated with bisphosphonates.^6^ Thus, a treatment that not only inhibits bone resorption but also stimulates bone formation may be desirable in the setting of severe disuse. Intermittent parathyroid hormone, or teriparatide, is the only clinically available treatment that promotes bone formation. However, intermittent PTH therapy also induces bone resorption and in some cases transient hypercalcemia, both of which are undesirable in the setting of disuse, in which negative calcium balance and high calcium excretion are common.^1^, ^7^ Given the profound bone loss seen in disuse, treatments that promote bone formation while limiting resorption would be ideal.

Previous work demonstrates that a soluble murine BMPR1A–fusion protein (mBMPR1A‐mFc) binds to BMP2/4 specifically and with high affinity.^8^ This in turn inhibits dickkopf‐1 (Dkk1) expression in osteoblasts to activate canonical Wnt signaling to promote bone formation. In addition, receptor activator of NF‐κB ligand (RANKL) expression in osteoblasts is reduced with the treatment, resulting in decreased bone resorption. Thus, treatment of normal and estrogen‐deficient mice with mBMPR1A‐mFc leads to increased bone mass and improved bone microarchitecture and strength, via increased bone formation and reduced bone resorption.^8^ In this study, we aimed to test the effects of mBMPR1A‐mFc on disuse‐induced bone loss using the murine hindlimb unloading model. We hypothesized that mBMPR1A‐mFc treatment would inhibit the bone loss seen in disuse through increased bone formation and reduced bone resorption compared to vehicle‐treated hindlimb unloaded (HLU) counterparts.

## Materials and Methods

### Overview of study design

Using baseline body mass and total body BMD to minimize group differences, we assigned 12‐week‐old female C57Bl/6J mice (Jackson Laboratory, Bar Harbor, ME, USA) to one of two loading groups: HLU or control (CON), and one of two treatment groups: mBMPR1A‐mFc or vehicle (VEH), resulting in four experimental groups (*n* = 10 each): (i) CON‐VEH; (ii) CON‐mBMPR1A‐mFc; (iii) HLU‐VEH; and (iv) HLU‐mBMPR1A‐mFc. Mice were exposed to HLU via tail suspension for 21 days as described.^9^ Measurements of body weight and subcutaneous injections of vehicle or mBMPR1A‐mFc (4.5 mg/kg; Acceleron Pharma, Cambridge, MA, USA) occurred twice per week. Mice were maintained on a 12‐hour/12‐hour light/dark cycle, had *ad libitum* access to standard laboratory rodent chow and water, and were euthanized by CO_2_ inhalation at the end of the experiment. All animal procedures were approved by and performed in accordance with the guidelines of the Institutional Animal Care and Use Committee (IACUC) at Beth Israel Deaconess Medical Center.

### In vivo BMD

We used peripheral dual‐energy X‐ray absorptiometry (pDXA) (PIXImusII; GE Lunar Corp., Madison, WI, USA) to measure total body (excluding the head region) and hindlimb BMD (g/cm^2^) in vivo, as described.^(9,10)^ Measurements were acquired at baseline and end of the study.

### Specimen harvesting and preparation

Mice were euthanized via CO_2_ inhalation. Serum was collected via cardiac puncture at euthanasia, following a 2‐hour fast. After being cleaned of its soft tissue, the right femur was wrapped in saline‐soaked gauze and stored at −20°C until mechanical testing. The left femur was prepared for histology and imaging in 70% ethanol at 4°C. Wet weight of the gastrocnemius and soleus muscles were obtained at end of study, and normalized to body weight.

### Histology and quantitative histomorphometry

Qualitative histologic analysis and quantitative static and dynamic histomorphometry were performed as described.^11^ Calcein (15 mg/kg) and demeclocycline (40 mg/kg) were injected intraperitoneally 8 and 2 days, respectively, prior to necropsy to allow for the investigation of bone formation rates. Histomorphometric measurements were performed on the secondary spongiosa of the distal femoral metaphysis using an OsteoMeasure morphometry system (Osteometrics, Atlanta, GA, USA). Static measurements in thin sections stained with Von Kossa/tetrachrome included osteoblast surface (Ob.S/BS, %) and osteoclast surface (Oc.S/BS, %). For dynamic histomorphometry, mineralizing surface per bone surface (MS/BS, %) and mineral apposition rate (MAR, μm/day) were measured in unstained sections under ultraviolet light, and used to calculate bone formation rate with a surface referent (BFR/BS, μm^3^/μm^2^/day). Terminology and units follow the recommendations of the Histomorphometry Nomenclature Committee of the American Society for Bone and Mineral Research (ASBMR).^12^

### Mechanical testing

Fresh frozen femurs were thawed and subjected to three‐point bending (Bose ElectroForce 3200 with 150 N load cell; Bose Corporation, Eden Prairie, MN, USA), with the anterior surface on the two lower support points spaced 8 mm apart and a constant displacement rate of 0.03 mm/s. Force‐displacement data were acquired at 30 Hz and used to determine maximum force (N), stiffness (N/mm), work to failure (N*mm), postyield displacement (mm), and estimated elastic modulus (MPa).

### Bone microarchitecture

We used high‐resolution microcomputed tomography (μCT40; Scanco Medical, Brüttisellen, Switzerland) to assess bone morphology and microarchitecture. Briefly, the distal femur and femoral midshaft regions were scanned using 70 kVp, 50 mAs, and 12‐μm isotropic voxel size. We assessed bone volume fraction (BV/TV, %), trabecular thickness (Tb.Th, mm), trabecular separation (Tb.Sp, mm), trabecular number (Tb.N, 1/mm), connectivity density (ConnD, 1/mm^3^), and structure model index (SMI) from a cancellous bone region that spanned 240 μm to 2040 μm distal of the growth plate in the femoral metaphysis region. We obtained the cancellous bone region using a semiautomated contouring program that separated it from the cortical bone. Cortical bone was analyzed from the metaphysis (surrounding the trabecular volume of interest) and from a 0.6‐mm‐long mid‐diaphyseal region. At the femoral midshaft, we assessed total cross sectional area, cortical bone area, and medullary area (TA, BA and MA, respectively, mm^2^); cortical bone area fraction (Ct.BA/TA, %), cortical thickness (Ct.Th, mm), porosity (Ct.Po, %) and minimum, maximum, and polar moments of inertia (Imin, Imax, and J, respectively, mm^4^). Bone was segmented from soft tissue using the same threshold for all groups, 267 mg hydroxyapatite (HA)/cm^3^ for trabecular and 682 mg HA/cm^3^ for cortical bone. Scanning and analyses adhered to published guidelines.^13^

### Statistical analysis

All data were checked for normality, and standard descriptive statistics were computed. Treatment effects were evaluated using analysis of variance (ANOVA) or repeated measures ANOVA for all continuous variables, followed by Tukey's honest significant difference (HSD) to test for differences between groups. Initial body mass was calculated as the average of body mass measurements made on day 0 and 1, whereas final body mass was the average of measurements made on day 18 or 19 and day 21. Differences were considered significant at *p* < 0.05. Data are reported as mean ± SE, unless noted.

## Results

### Body mass and muscle mass

Body mass was unchanged in the CON‐VEH and CON‐mBMPR1A‐mFc groups, whereas both HLU groups experienced a transient decrease in body mass compared to baseline in the first 3 days. However, only the HLU‐mBMPR1A‐mFc group experienced a small sustained decrease in body weight compared to baseline and to the normally‐loaded control group (–6% compared to CON‐VEH, *p* < 0.01 for both). As expected, HLU caused significant muscle atrophy in the hindlimb, as the gastrocnemius wet weight normalized to body weight was approximately 15% lower in the HLU‐VEH and HLU‐mBMPR1A‐mFc groups than in their respective controls (*p* < 0.01 for both). The soleus had greater atrophy, with a normalized wet weight that was about 40% lower in HLU mice than in fully loaded controls (*p* < 0.001 for both HLU groups). mBMPR1A‐mFc had no effect on muscle mass (Supporting Table  1).

### BMD

**Figure 1:**
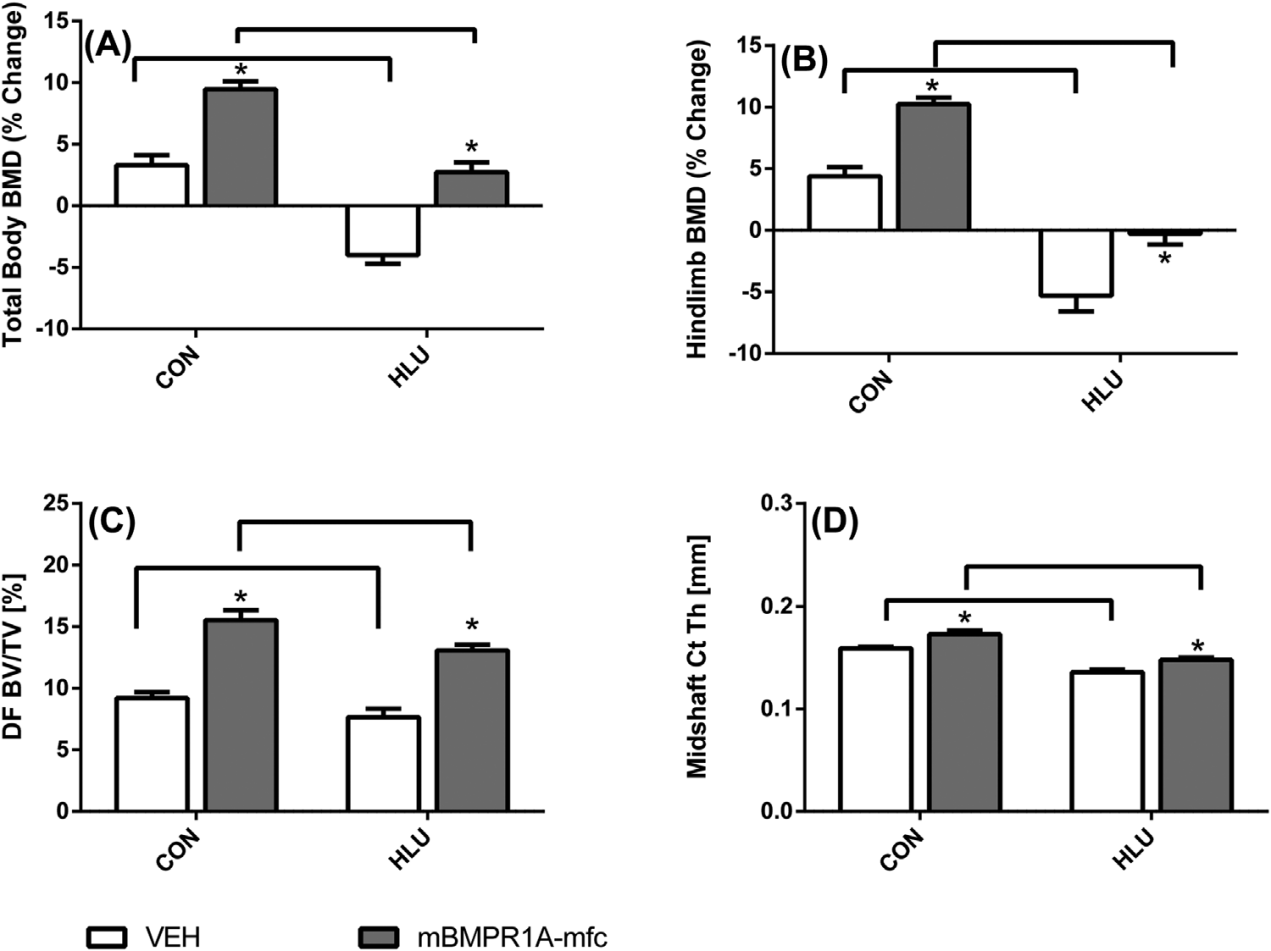
Effect of vehicle (open bars) and mBMPR1A‐mFC (gray bars) treatment on (*A*) total body BMD, (*B*) hindlimb BMD, (*C*) DF trabecular BV/TV, and (*D*) femoral midshaft cortical thickness in normally loaded (CON) and HLU mice. Horizontal bars show significant differences between CON and HLU mice within treatment group; *p < 0.05 for mBMPR1A‐mFc versus VEH within loading group. Error bars represent 1 SE. DF = distal femur; CON = control; HLU = hindlimb unloaded; VEH = vehicle.

Hindlimb and total body BMD both increased slightly from baseline in CON‐VEH, whereas BMD values declined significantly in the HLU‐VEH group at both sites (Fig. 1). In comparison, mBMPR1A‐mFc treatment increased BMD in normally loaded animals and either prevented BMD loss (hindlimb region) or increased BMD (total body) in the HLU group (Fig. 1). Specifically, hindlimb BMD increased 4.4% ± 0.8% in CON‐VEH and declined −5.3% ± 1.3% in the HLU‐VEH group (*p* < 0.01 versus baseline for both). Hindlimb BMD increased by 10.2% ± 0.6% in normally loaded controls treated with mBMPR1A‐mFc (*p* < 0.01 versus baseline and versus CON‐VEH), whereas hindlimb BMD was maintained in HLU mice treated with mBMPR1A‐mFc (0.3% ± 0.9%, *p* < 0.05 versus HLU‐VEH). Patterns for total body BMD were similar, except that treatment with mBMPR1A‐mFC in the HLU group led to significantly increased BMD versus baseline, and significantly higher BMD than the HLU‐VEH group (Fig. 1). The increase in total body BMD in mBMPR1A‐mFc treated animals was significantly greater in CON than HLU (*p*_interaction_ < 0.001).

### Bone microarchitecture

Overall, HLU worsened trabecular and cortical bone microarchitecture, whereas mBMPR1A‐mFC treatment improved bone microarchitecture in both CON and HLU groups compared to VEH (Table 1, Fig. 1). For example, compared to CON‐VEH, HLU‐VEH had 22% and 14% lower Tb.BV/TV and Tb.Th, respectively (all *p* < 0.05). In cortical bone, mid‐shaft cortical area was 15% smaller, cortical thickness was 14% thinner, and polar moment of inertia was 16% lower in HLU‐VEH compared to CON‐VEH (all *p* < 0.05). Trabecular architecture was markedly enhanced in both CON and HLU mice treated with mBMPR1A‐mFC compared to VEH‐treated mice, with 68% to 82% greater BV/TV, along with greater Tb.Th and connectivity density, and reduced Tb.Sp (all *p* < 0.05). In both CON and HLU groups, mBMPR1A‐mFc treatment also led to improvements in cortical bone microarchitecture at the mid‐diaphysis with 5% to 14% larger cortical bone area, 7% to 8% greater cortical bone area fraction, and 8% to 9% thicker cortical bone (all *p* < 0.05). These cortical changes appear to be due to endosteal bone apposition, as mice treated with mBMPR1A‐mFC had lower midshaft medullary area but similar total cross‐sectional area as VEH‐treated groups (Table 1).

**Table 1:** Effect of HLU and mBMPR1A‐mFC treatment on femoral trabecular and cortical bone microarchitecture, assessed by µCT

	Controls		HLU		ANOVA results		
Site	Vehicle (*n* = 10)	mBMPR1A‐mFc (*n* = 10)	Vehicle (*n* = 10)	mBMPR1A‐mFc (*n* = 10)	*p*_load_	*p*_treatment_	*p*_interaction_
Distal trabecular							
BV/TV (%)	9.20 ± 0.005	15.50 ± .008^a^	7.21 ± 0.008^b^	13.10 ± .005^a^,^b^	0.008	<	0.5
Tb.N (mm^–1^)	3.83 ± 0.09	4.04 ± 0.05	3.73 ± 0.06	3.99 ± 0.08^a^	0.4	0.003	0.9
Tb.Th (mm)	0.049 ± 0.001	0.064 ± 0.002^a^	0.045 ± 0.002^b^	0.055 ± 0.001^a^	0.009	<	0.04
Tb.Sp (mm)	0.256 ± 0.007	0.235 ± 0.004^a^	0.263 ± 0.005	0.238 ± 0.005^a^	0.5	<	1.0
ConnD (mm^–3^)	80 ± 7	104 ± 4^a^	65 ± 8	112 ± 7^a^	0.8	<	0.2
SMI	3.03 ± 0.08	2.57 ± 0.08^a^	3.15 ± 0.09	2.47 ± 0.05^a^	0.9	<	0.3
Midshaft cortical							
Tt.CSA (mm^2^)	1.62 ± 0.02	1.58 ± 0.02	1.57 ± 0.03	1.56 ±	0.1	0.3	0.5
Ct.BA (mm^2^)	0.66 ± 0.01	0.69 ± 0.01^a^	0.56 ± 0.01^b^	0.64 ± 0.01^a^,^b^	<	0.003	0.5
Ct.MA (mm^2^)	0.91 ± 0.014	0.83 ± 0.013^a^	0.99 ± 0.023^b^	0.88 ± 0.016^a^	<	0.0009	0.6
Ct.BA/TA (%)	40.9 ± 0.3	43.8 ± 0.6^a^	35.70 ± 0.4^b^	38.7 ± 0.6^a^,^b^	0.1	0.0195	0.8
Ct.Th (mm)	0.159 ± 0.002	0.173 ± 0.004^a^	0.136 ± 0.003^b^	0.148 ± 0.002^a^,^b^	<	<0.0001	0.7
Ct.TMD (mg HA/cm^3^)	1186 ± 3	1185 ± 4	1173 ± 5^b^	1184 ±	0.08	0.3	0.2
pMOI (mm^4^)	0.291 ± 0.008	0.288 ± 0.007	0.244 ± 0.009^b^	0.257 ± 0.008^b^	<0.0001	0.5	0.3
Ct.Po (%)	0.257 ± 0.012	0.259 ± 0.007	0.267 ± 0.017	0.257 ± 0.008	0.7	0.7	0.6

Values are mean ± SE.

BV/TV = trabecular bone volume; Tb.N = trabecular number; trabecular thickness; Tb.Sp = trabecular separation; ConnD = connectivity density; SMI = structure model index; Tt.CSA** = ** total cross‐sectional area; Ct.BA = cortical bone area; Ct.MA = cortical medullary area; Ct.BA/TA = cortical bone area fraction; Ct.Th = cortical thickness; Ct.TMD = cortical tissue mineral density; HA = hydroxyapatite; pMOI = polar moment of inertia; Ct.Po = cortical porosity.

^a^ *p* < 0.05 mBMPR1A‐mFc versus VEH within loading group.

^b^ *p* < 0.05 CON versus HLU within treatment group.

### Mid‐femoral biomechanics

Three‐point bending tests of the femur revealed that HLU‐VEH had lower bending stiffness (–25%) and maximum force (–23%) compared to CON‐VEH animals (*p* < 0.001, Table 2). In general, femoral biomechanical properties were not altered by mBMPR1A‐mFc treatment, except that HLU‐mBMPR1A‐mFC animals had greater maximum force (+18%, *p* = 0.006) compared to HLU‐VEH (Table 2). There were no significant differences in postyield displacement between the groups.

**Table 2:** Effect of unloading and mBMPR1A‐mFc on mid‐femoral biomechanics

	Controls		HLU		ANOVA Results		
	VEH (*n* = 10)	mBMPR1A‐mFc (*n* = 10)	VEH (*n* = 10)	mBMPR1A‐mFc (*n* = 10)	*p*_load_	*p*_treatment_	*p*_interaction_
Stiffness (N/mm)	91.1 ± 2.0	90.8 ± 2.5	68.5 ± 3.5^b^	76.3 ± 3.9^b^	<0.0001	0.4	0.2
Max force (N)	16.0 ± 0.6	15.8 ± 0.4	12.4 ± 0.5^b^	15.0 ± 0.7^a^	0.0008	0.09	0.01
Work to failure (N*mm)	9.55 ± 1.27	10.27 ± 1.39	8.40 ± 1.19	7.16 ± 0.96	0.08	0.8	0.4
Postyield displacement (mm)	0.90 ± 0.18	0.89 ± 0.17	1.15 ± 0.23	0.66 ± 0.14	0.1	0.2	0.4
Estimated Young's modulus (GPa)	10.57 ± 2.07	10.26 ± 0.89	10.01 ± 28.1	10.21 ± 15.76	0.5	0.9	0.6

Values are mean ± SE.

^a^ *p* < 0.05 mBMPR1A‐mFc versus VEH within loading group.

^b^ *p* < 0.05 CON versus HLU within treatment group.

### Histomorphometry

**Figure 2:**
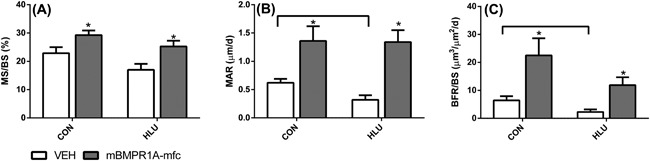
Effect of vehicle (open bars) and mBMPR1A‐mFC (gray bars) treatment on (*A*) MS/BS (%), (*B*) MAR (μm/day), and (*C*) BFR/BS (μm^3^/μm^2^/day) in normally loaded (CON) and HLU mice. Horizontal bars show significant differences between CON and HLU mice within treatment group; *p < 0.05 for mBMPR1A‐mFc versus VEH within loading group. Error bars represent 1 SE. CON = control; HLU = hindlimb unloaded; VEH = vehicle.

**Figure 3:**
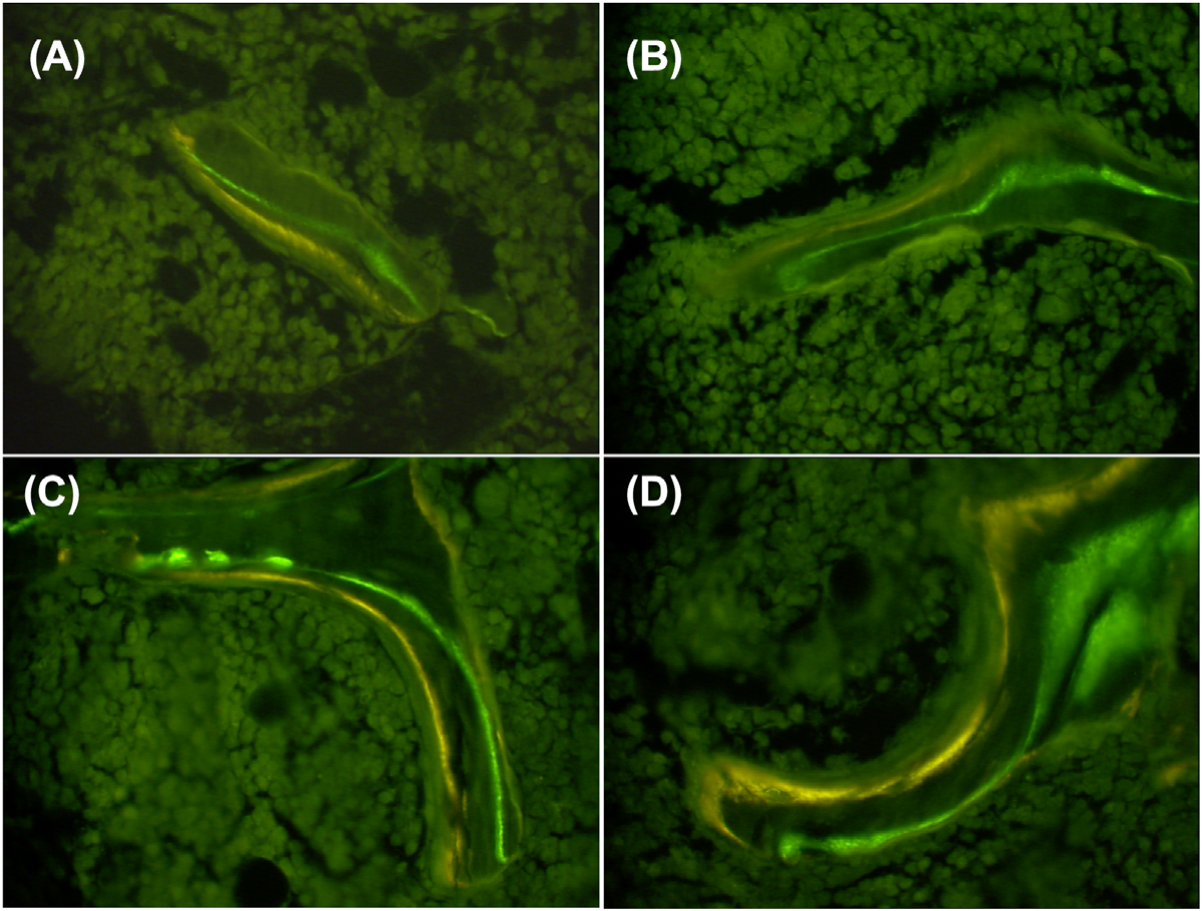
Representative image of fluorochrome labeling of trabecular bone surfaces in (*A*) Control‐Vehicle; (*B*) HLU‐Vehicle; (*C*) Control‐mBMPR1A‐mFc; and (*D*) HLU‐mBMPR1A‐mFc. Note the increased distance between labels in mice treated with mBMPR1A‐mFc, indicative of the increased mineral apposition rate. Images acquired under fluorescent light at magnification ×400. HLU = hindlimb unloaded.

Bone formation was decreased by exposure to HLU, as HLU‐VEH had 50% lower MAR (*p* < 0.01) and a trend for lower MS/BS (*p* = 0.09), leading to a 2.8‐fold lower bone formation rate (*p* < 0.01) compared to CON‐VEH (Fig. 2). HLU‐VEH also tended to have lower osteoblast surface (20% ± 3% in CON‐VEH versus 14% ± 4% in HLU‐VEH, *p* = 0.17) but had similar osteoclast surface (2.10% ± 0.33% in CON‐VEH versus 2.51% ± 0.41% in HLU‐VEH). Treatment with mBMPR1A‐mFC led to a markedly increased mineralizing surface and MAR, resulting in a 3.5‐fold and fivefold increase in bone formation rate in the CON (*p* = 0.01) and HLU (*p* = 0.02) animals, respectively (Fig. 3). Treatment with mBMPR1A‐mFC did not affect osteoblast surface, but led to a 60% reduction in osteoclast surface in both the CON (2.10% ± 0.76% in VEH versus 0.76% ± 0.19% in mBMPR1A‐mFC, *p* = 0.003) and HLU (2.58% ± 0.41% in VEH versus 1.03% ± 0.12% in mBMPR1A‐mFC, *p* = 0.004) groups.

## Discussion

In this study, we found that treatment of mice with a soluble mBMPR1A‐mFc fusion protein not only inhibited the bone loss due to HLU, but led to profoundly greater bone mass and strength compared to HLU‐VEH. This suggests that the anabolic effect of mBMPR1A‐mFC treatment was retained despite continuous HLU. Further, dynamic histomorphometry of the trabecular bone compartment demonstrated that increases in bone mass were due to increased bone formation indices and reduced osteoclast surface in both the normally‐loaded and unloaded mBMPR1A‐mFC‐treated mice. The increases in BMD, bone microarchitecture, and bone formation rate, and decreases in osteoclast surface seen in the mBMPR1A‐mFC‐treated animals were consistent with those previously reported by Baud'huin and colleagues^8^ in normal and ovariectomized mice.

As in prior reports,^9^, ^10^ HLU led to a pronounced decline in bone mass and deterioration of bone microarchitecture, accompanied by a marked decrease in bone formation indices. Previous studies in rodents report upregulation of the Wnt‐signaling antagonists *SOST* and *DKK1* following disuse via HLU or Botox injection.^14^, ^15^, ^16^ Further, increased levels of *Sost* and *DKK1* following unloading may also contribute to skeletal deterioration by enhancing osteoclastogenesis via increased levels of the pro‐resorptive cytokine RANKL and decreased levels of the RANKL decoy receptor, osteoprotegerin (OPG).^17^ mBMP1RA‐mFC treatment inhibits DKK1 expression and activates canonical Wnt signaling,^8^ which in turn prevents skeletal deterioration associated with HLU.

We showed that the negative effects of unloading were reversed by inhibition of BMP2/4 signaling via treatment with the soluble BMPR1A fusion protein, which led to increased bone mass and improved bone microarchitecture by increased bone formation rate and decreased osteoclast surface. This is consistent with prior work showing that inhibition of BMP2/4 signaling in the postnatal skeleton, either by treatment with mBMPR1A‐mFc or by conditional deletion of BMPR1A in osteoblasts, downregulates the expression of Wnt inhibitors *Sost* and *DKK1*.^8^, ^18^ This subsequent upregulation in Wnt signaling would be expected to increase bone formation and may explain the transient increase in osteoblast number seen with mBMPR1A‐mFc treatment or conditional deletion of BMPR1A in osteoblast lineage cells.^8^, ^19^ Inhibition of BMPR1A in osteoclasts may have also contributed to increased bone formation, because Okamoto and colleagues^20^ reported that conditional deletion of BMPR1A in differentiated osteoclasts led to increased bone formation and decreased osteoclast number and eroded surface in 8‐week‐old mice. We also observed lower osteoclast surface in mice treated with mBMPR1A‐mFc, an effect that might be mediated indirectly by effects of altered BMP2/4 signaling in osteoblasts or osteocytes, and/or by direct effects on osteoclasts. In support of indirect effects, Baud'huin and colleagues^8^ reported that inhibition of BMP2/4 signaling through administration of mBMPR1A‐mFc led to decreased serum levels of RANKL and increased levels of OPG. Also, deletion of BMPR1A in osteocytes decreased RANKL and increased OPG mRNA levels.^21^ Altogether, soluble BMPR1A‐Fc is a potent anabolic agent that promotes bone formation while inhibiting resorption.

Our study had several limitations, including the use of a single dosing regimen of mBMPR1A‐mFc and assessment of a single time point. Thus we may have missed early increase in osteoblast surface associated with mBMPR1A‐mFc, as reported by Baud'huin and colleagues^8^ and/or a transient increase in osteoclast surface due to unloading. Future dose‐ranging studies with additional time points would be helpful in defining the mechanisms that are responsible for the skeletal responses to mBMPR1A‐mFc.

These limitations notwithstanding, these results suggest that inhibiting signaling through the endogenous BMPR1A receptor by treatment with a soluble mBMPR1A‐mFc may be useful for maintenance of skeletal integrity in the setting of disuse osteoporosis as it increases bone formation and reduces bone resorption. Further work is needed to determine the optimal dose and delineate specific mechanisms underlying these observations.

## Disclosures

Dr. Bousein is a consultant for Acceleron Pharma. No other authors have conflicts of interest.

## Supporting information

Supporting Table S1.
