# Supplementary material for: Treatment With a Soluble Bone Morphogenetic Protein Type 1A Receptor (BMPR1A) Fusion Protein Increases Bone Mass and Bone Formation in Mice Subjected to Hindlimb Unloading
Source: JBMR Plus. 2017 Oct 9;1(2):66–72. doi: 10.1002/jbm4.10012 (PMC6124165; doi:10.1002/jbm4.10012)
Supplement: Supplementary file 1 — Supporting Table S1. [file JBM4-1-66-s001.docx]

**Supplemental Table 1**: Effect of HLU and mBMPR1A-mFC treatment on body mass, gastrocnemius, and soleus muscle mass (mean ± SEM).

|  | **Controls** | | **HLU** | | **ANOVA Results** | | |
| --- | --- | --- | --- | --- | --- | --- | --- |
| **Site** | Vehicle  n=10 | mBMPR1A-mFc  n=10 | Vehicle  n=10 | mBMPR1A-mFc  n=10 | p_load_ | p_treatment_ | P_interaction_ |
| Body mass (g) | 19.1 ± 0.33 | 18.8 ± 0.25 | 18.6 ± 0.25 | 18.0 ± 0.28 | 0.028 | 0.10 | 0.64 |
|  |  |  |  |  |  |  |  |
| *Gastrocnemius* |  |  |  |  |  |  |  |
| Muscle mass (mg) | 110 ± 1.9 | 107 ± 1.3 | 91.1 ± 1.6 | 87.1 ± 1.7 | <0.001 | 0.034 | 0.82 |
| Normalized muscle mass (mg/g) | 5.78 ± 0.05 | 5.71 ± 0.06 | 4.89 ± 0.04 | 4.84 ± 0.09 | <0.001 | 0.34 | 0.91 |
|  |  |  |  |  |  |  |  |
| *Soleus* |  |  |  |  |  |  |  |
| Muscle mass (mg) | 6.37 ± 0.16 | 6.21 ± 0.13 | 3.71 ± 0.12 | 3.66 ± 0.06 | <0.001 | 0.43 | 0.65 |
| Normalized muscle mass (mg/g) | 0.34 ± 0.006 | 0.33 ± 0.006 | 0.20 ± 0.004 | 0.20 ± 0.003 | <0.001 | 1.0 | 0.32 |
